# A non-inferiority study to compare daily fast-acting insulin versus twice a week slow-acting insulin–moderate diabetes mode^1^

**DOI:** 10.1590/s0102-865020200070000004

**Published:** 2020-08-05

**Authors:** Cristina Pires Camargo, Rafael Hori Nagamine Weschenfelder, Guilherme Moreira da Fonseca, Alexandre Agostinho da Cruz Sousa, Rolf Gemperli

**Affiliations:** I PhD, Division of Plastic Surgery, Hospital das Clínicas, Laboratory of Microsurgery and Plastic Surgery (LIM-04), Medical School, Universidade de São Paulo (USP), Brazil. Intellectual and scientific content of the study, interpretation of data, statistics analysis, manuscript writing, critical revision.; II Graduate student, Hospital das Clínicas, LIM-04, Medical School, USP, Sao Paulo-SP, Brazil. Acquisition, analysis and interpretation of data; technical procedures.; III PhD, Division of Plastic Surgery, Hospital das Clínicas, LIM-04, Medical School, USP, Sao Paulo-SP, Brazil. Intellectual and scientific content of the study, interpretation of data, critical revision.

**Keywords:** Diabetes mellitus, Insulin, Rats

## Abstract

**Purpose:**

Given the high prevalence of diabetes (D), several animal models have been analyzed. In the literature, most of the animal models have studied severe D. However, in clinical practice, most patients have moderate disease. Therefore, the present study aimed to describe a moderate D condition.

**Methods:**

We analyzed 20 Wistar rats, age eight-weeks, weight between 200g-250g. All animals received an intravenous injection of Streptozotocin (55mg/kg weight). On the 15th day after D induction, the animals were divided into two groups: Group I – animals receiving a single daily dose of fast-acting insulin (FAIG) NPH (1UI,SC) for partial glycemic control, and Group II - animals receiving slow-acting insulin(SAIG) twice a week. We measured glycemia, weight, and adverse events every week during two months.

**Results:**

Of the total of animals analyzed in the study, three animals died in the FAIG and two animals died in the SAIG. Regarding the glycemic level, results were 339.5 ± 125.4mg/dL (95CI 302.3402 to 376.6842) in the FAIG, and 367.8 ± 66.1mg/dL (95IC 333.7607 to 401.8978) in the SAIG. There was no difference between groups as to weight during the study.

**Conclusion:**

The use of slow-acting-insulin is not inferior to the use of fast-acting-insulin in the management of partially insulin-controlled moderate diabetes in rats.

## Introduction

Diabetes has a high prevalence in the world population. In Brazil, the prevalence of diabetes is around 12%^1^.

Because of this high prevalence, pre-clinical trials are attractive to study therapeutic strategies to control diabetes^1-3^.

Several studies have induced diabetes using a single injection of streptozotocin.

After inducing diabetes, some weeks are required for diabetes to cause macro and microangiopathy^5-7^.

Diabetes affects endothelial function, leading to subclinical inflammation. The inflammation status associated with increased peripheral insulin resistance, hyperglycemia, and oxidative stress cause vasoconstriction and deficit in blood supply to the surgical wound. The possible mechanisms that lead to decreased blood supply are the reduction of nitrous oxide production that activates the sugar polyol pathway, producing “AGEs” (advanced glycation end products). AGEs increase the production of protein kinase, responsible for vasoconstriction. In addition, diabetes decreases the rheological properties of the blood, causing platelet aggregation and decreasing the migration of epidermal cells required for wound regeneration, thus contributing to the failure of the healing process^6,7^.

The criticism to this model is that these animals show a severe level of diabetes, and after ten weeks most of the animals die due to diabetes complications. The diabetes model, however, is efficacious; nevertheless, this condition is not representative of the majority of patients, who present a moderate level of diabetes. In this sense, it is interesting to standardize a moderate diabetes condition in the animal model.^1^

In the literature, the use of type I diabetes-inducing agents, e.g. streptozotocin, is well-founded. However, when using this method in rats, the survival of these animals is limited to around ten weeks. It would be interesting to extend this survival by using insulin to study the effect of drugs that can alter the viability of the dorsal skin flap in animals that mimic chronic type I diabetes with partial glycemic control. This scenario reflects what we find in daily practice. Several studies have reported the daily administration of fast-acting insulin to mimic moderate chronic diabetes^6,7^. There are some indications^7^ that the use of slow duration insulin can promote the same clinical situation with the advantage of subjecting the animal to greater stress (due to daily handling).

This project proposes a non-inferiority study design to analyze the effect of different types of insulin on the glycemic curve of diabetic rats in the period of nine weeks.

## Methods

This study was approved by CEUA (66/15) and followed CONCEA guidelines.

We analyzed twenty-eight-week-old male Wistar rats weighing 200-250g. All animals were kept in a vivarium with a day / night cycle, and water and food “ad libitum”. Diabetes was induced in the animals, and after six weeks they were divided into two groups:

**Group I** - Animals that received fast-acting NPH insulin (1UI, subcutaneous) once daily for partial glycemic control,

**Group II** - Animals that received slow-acting insulin (1UI, subcutaneous) twice a week.

The primary endpoint was glycemia at week nine. Secondary endpoints were: week glycemia measurement, weight at week nine, mortality.

### *Diabetes induction*

Rats were fasted for eight hours. Under inhalation anesthesia, blood was collected from the tail vein to measure blood glucose before the streptozotocin injection.

All the animals received an intravenous injection (penile vein) of 1% Streptozotocin, at a dose of 55mg/kg, diluted in PBS, pH = 4.5.

After 24h of the streptozotocin injection, a new glycemia was measured under general anesthesia. Animals with blood glucose greater than 200mg/dl were considered diabetic.

The animals were observed for another six weeks before starting insulin treatment.

### *Insulin injection*

The animals allocated to the fast-acting insulin group received Novolin^®^, (recombinant DNA origin, Insulin NPH 100uL/mL, Novo Nordisk, Brazil), 0.5-0.7U/Kg, a single daily dose (Human) at 4:00 pm

The animals allocated to the slow-acting insulin group received Lantus^®^ (insulin glargine, recombinant DNA origin, Sanofi Aventis, Brazil), 0.5-0.7U/Kg, single dose twice a week at 4:00 pm.

The animal’s blood glucose determined the total units of insulin. This partial blood glycemic treatment aimed to keep blood glycose around 300-380mg/dL^7^.

### *Analysis of glycemia and weight*

Glycemia was measured weekly by puncture in the tail vein of the animal using specific equipment for this purpose.

Glycemia was measured once a week for nine weeks. The animal’s tail was punctured, and a drop was placed on a blood glucose meter (Accu-check, Roche, Brazil). All animals were weighed weekly to plot on a graph.

### *Euthanasia*

Nine weeks after the beginning of the procedure, the animals were euthanized by an intraperitoneal injection of Pentobarbital (100mg/Kg).

After euthanasia, the animals received the necessary measures for their disposal according to the Waste Disposal Guideline in the FMUSP-HC System, which follows Resolution number 306, of December 7, 2004, of the National Health Surveillance Agency, and the Resolution of the National Environment Council – CONAMA, number 358, of April 29, 2005.

### *Statistical analysis*

Data were analyzed using descriptive statistics in which the mean, median, standard deviation were calculated. A non-parametric comparison test was performed to compare the groups.

This study is a non-inferiority study, so the established non-inferiority margin was 10% (350mg/dL) below the mean glycemia reported in the literature^7^.

We used the STATA v14 (StataCorp. 2015. Stata Statistical Software: Release 14. College Station, TX: StataCorp LP) program for statistical analysis.

### *Sample size calculation*

According to literature data, we calculated the sample size. Considering alpha p of 5% and power of 80%, the sample size per group was 10 animals.

## Results

Twenty-four hours after the Streptozotocin (STZ) injection all animals became diabetic (blood glucose> 200mg/dL). After a six-week period, 100% of the animals remained diabetic and study measurements began.

Of the total animals analyzed in the study, three animals died in the fast-acting insulin group, and two animals died in the slow-acting insulin group.

At 9-weeks after STZ, mean glycemia in the groups was around 350mg/dl as proposed in our hypothesis, and as described in Table 1.

**Table 1:** Glycemia levels at nine weeks in groups (mean, standard deviation and 95% confidence interval).

Insulin group	Mean ± SD (mg/dL)	95% CI
fast-acting	339.5 ± 125.4	302.3402 376.6842
slow-acting	367.8 ± 66.1	333.7607 401.8978

95% IC-95% confidence interval

**Figure 1:**
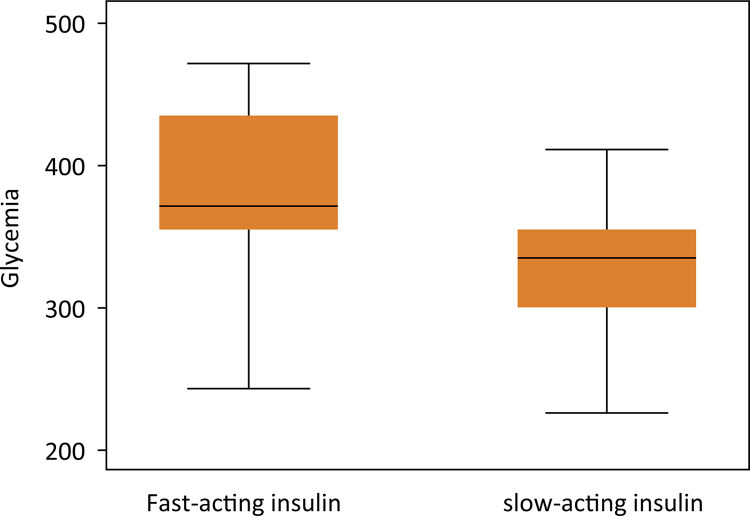
Box-plot graph: glycemia levels at nine weeks.

There was no difference in glycemia values measured between groups over nine weeks (p = 0.5660). The confidence interval of the fast-acting insulin group and slow-acting insulin complied with the established margin of non-inferiority (Table 1, Fig. 1).

**Figure 2:**
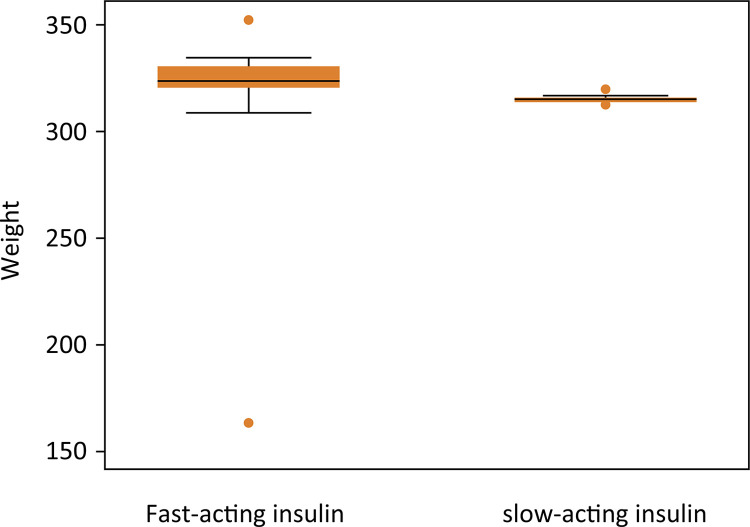
Box-plot graph of weight measurements at nine weeks.

There was no difference in weights between the groups analyzed (p = 0.4138) (Table 2, Fig. 2).

**Table 2:** Weight in groups (mean, standard deviation and 95% confidence interval).

Insulin group	Mean ± SD (mg/dL)	95% IC
Fast-acting	326.0 ± 3.9	318.0821 333.9179
Slow-acting	320.9 ± 5.8	308.5616 331.9579

95% IC-95% confidence interval

## Discussion

Our study showed that using slow-acting insulin is not inferior to fast-acting insulin for partially controlling glycemia in diabetic rats induced by streptozotocin. This finding is relevant to create an experimental model of partially controlled chronic diabetes, because of the high prevalence of individuals with moderate diabetes in our daily clinical practice^6,8^. According to literature data, there are several animal models to induce diabetes.

One possibility are genetically modified animals (WDF / TA-FA, Zucker) that can be adopted as models of diabetes-associated with metabolic syndrome.^9^ Although the model is valid, there are high costs and reproducibility is difficult.

Another procedure to cause diabetes in animals is pancreatectomy; the surgery removes the pancreas and consequently causes severe diabetes. The disadvantage of the method is the high mortality of the animals undergoing the procedure.

Diabetes can also be induced by gene therapy and drugs. Gene therapy represents high costs and complexity. On the other hand, pharmacological therapy is generally adopted due to its ease of use and low cost^2,11^.

Among the drugs available on the market, alloxan and streptozotocin are the most used. The percentage of animals induced into diabetic status after an intravenous streptozotocin injection is higher than with the use of alloxan. In this study, there was a 100% success rate in inducing diabetes 24 hours after the intravenous STZ injection^9-11^.

When drugs induce diabetes, most experiments only investigate the progression of the disease. The interval from induction to the experimental part of the study has ranged from 6-8 weeks^3,11^. After this period, animals develop severe metabolic disorders, and these models express severe diabetes. As a criticism to this model, analysis of severe diabetes does not reflect the practice, that is, studying methods to increase viability of surgical flaps in diabetic patients who partially control blood glucose.

In this sense, a chronic model of diabetes partially controlled by insulin to mimic a large part of the population is desirable^2^. Several authors have analyzed the use of a slow-release insulin pump implanted in the subcutaneous tissue of diabetic animal models^17^. Although this model is relevant, costs and complexity of handling limit its use^14^.

Another model described in the literature was the use of a biodegradable matrix embedded in insulin implanted in the subcutaneous tissue of animal models, the main disadvantage being the lack of control of units released during the deterioration of these implants^6,15^.

Given these factors, subcutaneous insulin injection would be the best option for secure handling, low cost, and control of the administered dose unit. Many studies use subcutaneous injections of fast-acting insulin daily. This technique involves daily injections in the afternoon. Fast-acting insulin has an onset of action of 30 minutes and a peak of action between 2 and 4 hours and activity of up to 8 hours. Slow-acting insulin has an onset of 1-2 hours and an action of up to 14h^8,17^.

The idea of using slow-acting insulin for our animal model was based on reducing animals’ stress, as insulin was injected only twice a week and the injection is easily performed.

The groups assessed demonstrated that the use of fast-acting insulin promoted considerably higher variability in the glycemic level of the animals in this group, while animals treated with slow-acting insulin showed stable blood glycemic levels.

The fluctuation in glycemia level observed in the fast-acting insulin group may be a factor for the higher mortality rate observed in this group. Another factor that corroborates using slow-acting insulin would be low weight variability over the study period.

As a limitation of this study, we did not measure serum levels of other catabolic hormones such as cortisol. Perhaps less animal handling would be associated with a lower level of cortisol, compared to animals that are handled daily. As less handling decreases animal stress, it can consequently contribute to better animal welfare and lower effects of other hormones on animal metabolism.

## Conclusion

Using slow-acting insulin is not inferior to using fast-acting insulin in the maintenance of partially insulin-controlled moderate diabetes in rats.

## Footnotes

1 Research performed at Laboratório de Investigação Médica de Microcirurgia e Cirurgia Plástica (LIM04), Medical School, Universidade de São Paulo (USP), Brazil.
